# Synthetic biology of fungal natural products

**DOI:** 10.3389/fmicb.2015.00775

**Published:** 2015-07-30

**Authors:** Derek J. Mattern, Vito Valiante, Shiela E. Unkles, Axel A. Brakhage

**Affiliations:** ^1^Department of Molecular and Applied Microbiology, Leibniz Institute for Natural Product Research and Infection Biology – Hans Knöll Institute, Jena, Germany; ^2^Institute for Microbiology, Friedrich Schiller University, Jena, Germany; ^3^Leibniz Junior Research Group “Biobricks of Microbial Natural Product Syntheses”, Jena, Germany; ^4^School of Biology, Biomedical Sciences Research Complex, University of St Andrews, St Andrews, UK

**Keywords:** synthetic biology, fungal natural products, regulation of natural products, heterologous expression, engineering of biosynthetic enzymes

## Abstract

Synthetic biology is an ever-expanding field in science, also encompassing the research area of fungal natural product (NP) discovery and production. Until now, different aspects of synthetic biology have been covered in fungal NP studies from the manipulation of different regulatory elements and heterologous expression of biosynthetic pathways to the engineering of different multidomain biosynthetic enzymes such as polyketide synthases or non-ribosomal peptide synthetases. The following review will cover some of the exemplary studies of synthetic biology in filamentous fungi showing the capacity of these eukaryotes to be used as model organisms in the field. From the vast array of different NPs produced to the ease for genetic manipulation, filamentous fungi have proven to be an invaluable source for the further development of synthetic biology tools.

## Introduction

Since the onset of synthetic biology in the early 1990s to the explosion of genomics data in the early 2000s, a new discipline has emerged. The exact definition of synthetic biology is still an intriguing question to many. One of the first uses of this concept was with the discovery of restriction enzymes with the Nobel Prize in Physiology and Medicine in 1978 (Szybalski and Skalka, 1978). The engineering of microorganisms for the production of compounds has been followed for a long time. Now, based on the advent of genome-based methods synthetic biology is a rigorous engineering discipline to create, control and program cellular behavior. In this understanding the end goal is to ultimately be able to engineer a system/organism to perform how we want it to perform (Cameron et al., 2014). This concept has also been extensively used in the discovery and understanding of natural product (NP) research in different microorganisms. Microorganisms are a treasure trove of NPs and the potential has just been realized with the beginning of the genomics era (Brakhage, 2013). Concerning the synthetic biology aspects of NP research much has also been done in the model organism *Saccharomyces cerevisiae* because of its vast toolkit in making different genomic manipulations (Siddiqui et al., 2012). But as will be discussed in the following review, filamentous fungi also hold the potential as organisms that can be used in the field of synthetic biology to essentially control the production of NPs. Whether looking at it from the perspective of novel products or optimizing production conditions of known bioactive molecules, fungi have proven their own potential as work horses in the field of NP research. Different aspects will be discussed in this review from the manipulation of different regulatory/enzyme elements to the use of fungal NP gene clusters or the fungus itself in heterologous expression.

## Manipulation of Natural Product Regulatory Elements

**FIGURE 1:**
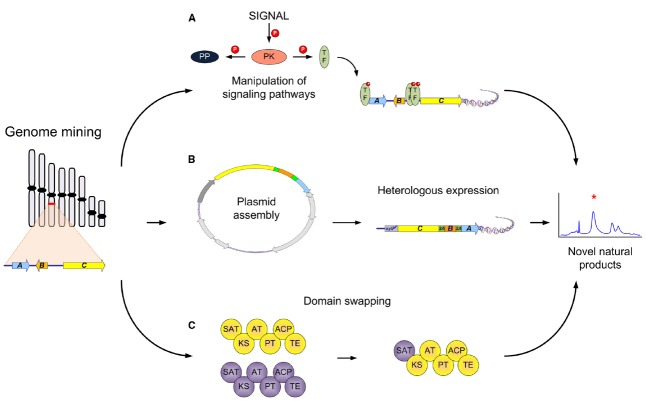
**Synthetic biology strategies for natural product production in filamentous fungi.** Different strategies that could be used to activate computationally identified gene clusters: **(A)** Manipulation of different elements of signal transduction pathways leading to novel or different regulation of NPs. The activation (by phosphorylation) of transcription factors (TF), global or NP gene cluster specific, can be achieved by specific stimuli (signal), engineering of protein kinases (PK), or deletion of signal repressors such as protein phosphatases (PP); **(B)** Heterologous expression of known or unknown NP gene clusters in an amenable host to achieve production of novel/bioactive NPs. This includes the assembly of a plasmid containing the necessary/putative genes involved and then transformation and heterologous expression to achieve production; **(C)** Domain swapping of different parts of multidomain biosynthetic enzymes such as PKSs. Different aspects such as the swapping of the C or N terminal domains have been accomplished for production of novel NPs.

The dawn of genome sequencing has unveiled the capacity microorganisms hold in order to create different NPs. This is also true for filamentous fungi, where in some species close to 80 different gene clusters have been predicted (Inglis et al., 2013). Since most of these potential gene clusters are not expressed under standard laboratory conditions, different methods have been used in order to activate these silent/cryptic gene clusters (Brakhage, 2013). Most of the examples entail manipulation of the different regulatory elements in the gene cluster or global regulators (LaeA or VelA; Yin and Keller, 2011; Sarikaya-Bayram et al., 2015). Additionally, the manipulation of different signaling pathways, such as protein kinases (PK) and protein phosphatases (PP), can strongly influence the expression of NP gene clusters (Figure 1A Valiante et al., 2015).

One of the first examples entailed overexpressing a gene cluster specific transcription factor to activate the production of a novel compound. This was done in the model organism *Aspergillus nidulans* and resulted in the production of the novel aspyridones (Bergmann et al., 2007). This technique is of particular interest because many different promoters can be utilized from constitutive to inducible expression. Examples can range from constitutive promoters; such as the *gpdA* (glyceraldehyde-3-phosphate dehydrogenase; Punt et al., 1990) or the *otef* synthetic promoter modified from the original *tef* promoter (Spellig et al., 1996) to inducible promoters such as the *alcA* (alcohol dehydrogenase I) promoter, the *pXyl* xylose inducible promoter (Zadra et al., 2000) or the tetracycline-inducible promoter system (Helmschrott et al., 2013; Macheleidt et al., 2015). These are just to name a few, but new promoters are constantly being adapted and studied to optimize different aspects of regulation. Additionally, other approaches for the activation of silent clusters can also result from the cocultivation of different microorganisms. Two examples of cocultivations of *Streptomyces rapamycinicus* with *A. nidulans* and *A. fumigatus*, where in both cases silent NP clusters were activated and new NP produced (Schroeckh et al., 2009; König et al., 2013). From the synthetic biology perspective this interaction has also been studied and found that chromatin modifications play a key role in this interaction. In a groundbreaking study by Nützmann et al. (2011) it was found that the bacterium, *S. rapamycinicus*, actually activates a histone acetyltransferase (HAT) in the fungus resulting in the activation of this silent gene cluster. This HAT, GcnE, part of the Saga/Ada complex was also found to play a role in the regulation of different NPs and in a further study it was shown that when specific amino acids on the histone tail of H3 were replaced mimicking acetylation or non-acetylation it affected the production of different NPs (Nützmann et al., 2013). This work shows the potential of manipulating the chromatin landscape to activate/repress different aspects of secondary metabolism. Other examples using chromatin modifications for the regulation of different NPs have been previously reviewed (Netzker et al., 2015).

Besides the activation of different NPs, there is also a growing field encompassing the compartmentalization/localization of NPs in filamentous fungi. Early studies focused on the peroxisome and penicillin biosynthesis. Peroxisomes are specialized organelles in eukaryotes involved in a variety of functions from primary, β-oxidation of fatty acids, to secondary metabolism, where they play a role in penicillin biosynthesis (Bartoszewska et al., 2011). Interestingly, the first two steps of penicillin biosynthesis take place in the cytosol, but the last step occurs within the peroxisome (Spröte et al., 2009). Different studies have shown synthetic biology techniques manipulating the peroxisome number (Opalinski et al., 2011) to localizing other genes from the penicillin pathway into the peroxisome (Herr and Fischer, 2014), where both resulted in an increase in penicillin production. Besides penicillin, other metabolites have also been shown to be localized to the peroxisomes such as siderophores in *A. fumigatus* and aflatoxin biosynthesis in *A. flavus* (Reverberi et al., 2012; Gründlinger et al., 2013). The compartmentalization of NP products has been extensively reviewed by other groups (Kistler and Broz, 2015), but the potential to use this knowledge and apply it to synthetic biology is endless. Keeping the precursors and enzymes in tightly controlled compartments is a dream that many scientists envision and to better understand this would help in increasing product yields and may be useful in the discovery of novel NPs.

Two other examples that epitomize the use of different regulatory elements for the production of NPs in filamentous fungi are from two different genera, *Aspergillus* and *Fusarium*. In both examples they established a novel fungal expression system using a promoter and an activator from an NP gene cluster. The first example with the equisetin biosynthetic pathway from *Fusarium heterosporum* utilized the native promoter from the polyketide synthase (PKS) gene and overexpression of the transcription factor in the native host. The native promoter was then used with an unknown PKS that was found to produce the antituberculosis agent pyrrolocin (Kakule et al., 2014a). This method is very beneficial when using the host *F. heterosporum*; while the study of Gressler et al. (2015) is a good example using different fungal hosts. In this study the authors assembled a ready-to-use plasmid where simple insertion of genes of interest can be used under the control of the constitutive promoter and governing regulatory gene that is also present in the plasmid. The system is very similar to the study from *F. heterosporum*, but this study uses *Aspergillus terreus* and the terrein NP cluster. This cluster in the native host produces a significant amount of terrein (>1.1 g/L), thus creating a fungal expression system with the native promoter and transcriptional activator is advantageous (Gressler et al., 2015). These two examples, both using regulatory elements to help increase/express NPs, whether novel or for industrial purposes, are good examples of coupling production with regulation in a heterologous host.

## Fungal Heterologous Expression Systems

The use of heterologous expression systems is a major topic in the study of fungal synthetic biology, because it not only allows one to study fungal enzymes/pathways in different hosts, but also shows the capacity of fungi to be the heterologous host. This topic of fungi as heterologous expression systems has been reviewed quite extensively in earlier review articles (Schümann and Hertweck, 2006; Lubertozzi and Keasling, 2009) and a more recent article from the Mortensen lab (Anyaogu and Mortensen, 2015), but in this work, we will highlight a few major advances in heterologous expression regarding synthetic biology. From one of the first attempts of heterologous expression in 1990 of expressing the penicillin biosynthetic gene cluster (Smith et al., 1990) many other studies have shown that fungal biosynthetic enzymes can be a valuable tool for the production of NPs. But the question remains as to which host will be used. There are many studies showing fungal NP enzymes in various hosts from different prokaryotes, such as *Escherichia coli*, to different eukaryotes, such as *S. cerevisiae* (Kealey et al., 1998) to even higher eukaryotes like the tobacco plant *Nicotiana tabacum* (Yalpani et al., 2001). One of the most promising studies to date a non filamentous fungal host was done in the yeast *Pichia pastoris*, which is also known for its high production of heterologous proteins (Cereghino and Cregg, 2000). In this particular study the *A. terreus* 6-methylsalicylic acid (6-MSA) synthase, which is a PKS, was coexpressed with the *A. nidulans* phosphopantetheinyl transferase (PPTase) gene and resulted in yields of 6-MSA as high as 2.2 g/L (Gao et al., 2013). This was the highest yield reported thus far from all the studies listed above, which also expressed the PKS for 6-MSA. But a lot of effort has also been put in optimizing *S. cerevisiae* for the production of fungal NPs. A recent review covers the entire field along with the use of fungal enzymes in *S. cerevisiae* (Siddiqui et al., 2012). One particularly interesting example in *S. cerevisiae* was with the pathway for rubrofusarin, where four different enzymes from two different filamentous fungi were expressed. Rubrofusarin biosynthesis is of interest because the intermediates are branch points of pigmented compounds in the spores of many different fungi (Rugbjerg et al., 2013).

In most cases when heterologously expressing fungal NP enzymes/pathways using a host that is from the same species results in a stronger likelihood of a positive result. Moreover, using a host that already possesses the ability of producing NPs will also improve the chances for success. Many different filamentous fungi have been used and shown to be ample hosts for heterologous expression of NPs. The most prominent thus far being *Aspergillus oryzae* (Sakai et al., 2008, 2012; Heneghan et al., 2010) and *Aspergillus niger* which is used as an industrial production strain for the synthesis of different organic acids, such as citric acid (Pel et al., 2007), along with NP production (Richter et al., 2014).

Heterologous expression can also be used in the discovery of novel NPs. Examples of this include coexpression of different genes to get novel prenylated cyclic dipeptides (Wunsch et al., 2015) to the combination of different ergot alkaloid biosynthetic genes to get novel products (Robinson and Panaccione, 2014). Other examples have been from other fungi where genetic manipulation is still not established but their genomic sequences are accessible. This being the case of the expression of a NP gene cluster from dermatophytes into the model organism *A. nidulans* and different PKS genes from *Talaromyces stipitatus* in *A. oryzae*, both resulting in new products (Yin et al., 2013; Hashimoto et al., 2015). These are just a few different ways in which heterologous expression can be useful especially in eukaryotes.

In the genomic era with all the different NP gene clusters uncovered there are a couple of important things to consider when utilizing heterologous expression. One is the actual detection of the product once transformed into the heterologous host. This could be one downfall when using a related species of filamentous fungi, because of their complex NP metabolome. Thus, making *S. cerevisiae* a frequently chosen host in the hunt for novel products, because of its lack of NP production. But there are a number of drawbacks resulting from just the fact that *S. cerevisiae* is not a natural host for NP production, e.g., the lack of PPTase genes or, possibly, the lack of precursors for NPs. But one of the best examples of this was done in *A. nidulans* where a very efficient heterologous expression host was developed. Essentially, many of the major NP clusters that could be highly detected were deleted, leaving the wild-type strain to have an almost “clean” background, which allowed for the easy detection of heterologously expressed clusters (Chiang et al., 2013). Additionally, one must also plan on how to assemble the genes/pathway before transformation into a heterologous host. This is in particular necessary when the cluster of desire lacks a pathway-specific transcription factor and thus overexpression of a transcription factor gene and thus expression of all cluster genes cannot be easily achieved.

Some classical ways can be done by simple PCR (Nielsen et al., 2013), but in one recent example a novel strategy for the bioprospecting of NPs was exemplified. In this example a plasmid consisting of genes encoding for the biosynthesis of penicillin were assembled by TAR cloning in *S. cerevisiae* and then transformed into an *A. nidulans* strain that is a non-producer of penicillin. Interestingly, in this case only one plasmid was needed containing every gene necessary for the production and once incorporated was also designed to be under the control of a single inducible promoter. This in turn is important because of the regulation of an entire cluster using one promoter that once transcribed would be present as a polycistronic mRNA. In addition when designing the original plasmid, between each individual gene, 2A viral peptides were used to separate them, which when translated by the ribosome cleaved each individual protein resulting in the production of penicillin (Unkles et al., 2014). This strategy incorporates many different techniques into the field of filamentous fungi synthetic biology from a novel expression system to the use of heterologous expression (Figure 1B). This technique exemplifies the reach we can achieve with synthetic biology and the control of NP production.

## Engineering of Fungal Biosynthetic Enzymes

Another aspect of synthetic biology entails the engineering of different biosynthetic enzymes, with a particular emphasis on the major players in NP biosynthesis, which are the multidomain PKSs and non-ribosomal peptide synthetases (NRPSs). Up until now, much work has been done to understand the mechanisms of these enzymes in both fungi and bacteria (Crawford and Townsend, 2010; Williams, 2013; Hertweck, 2015). A lot of work has also been performed in understanding these important biosynthetic enzymes in eukaryotes especially in filamentous fungi. Interestingly, in these organisms, type I-like PKSs are also present, but they work in an iterative way, repeating specific biosynthetic steps cyclically. Until now, we still cannot understand how these processes are regulated, and what are the “criteria” that drive the exact numbering of the different steps. This makes it almost impossible to make a computational prediction of the polyketide product, which can be sometimes only deduced based on the similarities with previously described enzymes (Hertweck, 2009; Chiang et al., 2010). Thus, much of the work up until now has been on achieving a better understanding of just how these particular enzymes function and with this understanding a better rationale can be used to engineer them. But of course these first studies have shown much potential for the future and use of these biosynthetic machines for the creation of novel compounds. Additionally, different and novel domains have been discovered in non-reducing PKS (NR-PKS) such as the starter unit acyl transacylase (SAT; Crawford et al., 2006) to the product template domain (PT; Crawford et al., 2008). Also, obtaining a crystal structure of these proteins is very difficult because of their insolubility, their size and apparent flexibility, but in the case with the PT domain from the NR-PKS from aflatoxin biosynthesis it was shown and gave even further insight into the core structure of the protein (Crawford et al., 2009). These studies were ground breaking for further studies on NR-PKS, where further manipulation could be completed by domain swapping of C or N terminal domains with different NR-PKS (Vagstad et al., 2013; Liu et al., 2014; Newman et al., 2014). With the domain swapping of the different NR-PKSs it really showed that a need for an “intimate” knowledge of the workings of these complex enzymes is necessary.

Besides the work on NR-PKS there has also been a lot of work on highly reducing PKS (HR-PKS) in filamentous fungi. These PKSs are also of interest because they come in different modes of reduction leading to a wide range of chemical diversity. Early studies also focused on the understanding of these complex enzymes and especially on the PKS responsible for the statin, lovastatin (Ma et al., 2009), the mycotoxin fumonisin (Zhu et al., 2006, 2007) and the protein transport inhibitor brefeldin (Zabala et al., 2014). The brefeldin study also demonstrated the need to consider the different tailoring enzymes necessary for the final product and specific product release. The PKS only creates the core structure, while different tailoring enzymes make additional modifications. This also represents a potential for the manipulation of different tailoring enzymes, such as cytochrome P450s (Podust and Sherman, 2012) or others, that may appear more frequently in the future. There have also been some success stories concerning the swapping of domains and in turn the discovery of novel products. Two recent studies one with an NR-PKS, with asperfuranone biosynthesis (Liu et al., 2011), and the other an HR-PKS-NRPS hybrid, from tenellin and desmethylbassianin (Fisch et al., 2011) successfully made domain swaps with the HR-PKS portion, which resulted in “unnatural” NPs. Additionally, other studies have focused on PKS-NRPS hybrids in order to understand how these different components communicate (Xu et al., 2010). In one study, Kakule et al. (2014b) utilized 57 different combinations with 34 different module switches of various fungal PKS-NRPS hybrids to yield six novel compounds. In filamentous fungi, only few studies have yet been devoted to NRPS engineering. Prior studies are mostly seen in bacteria (Calcott and Ackerley, 2014), but the potential in fungi is still there for further discovery. The engineering of fungal biosynthetic enzymes such as PKS or NRPS present a platform for the discovery of novel chemical derivatives that could hold the potential for new bioactive compounds (Figure 1C). With continuing work on the understanding of this complex biosynthetic machinery, hopefully one day we can successfully design novel combinations to get products of desired complexity.

## Perspectives

Synthetic biology for the discovery and understanding of fungal NPs is an ever-evolving field. With the advancement of the different techniques presented herein the possibilities are endless for the production of novel/bioactive NPs. Additionally, with the rapid discovery in different genome editing and genetic manipulation tools such as the bacterial derived CRISPR-Cas9 system (Doudna and Charpentier, 2014), these could also be applied to filamentous fungi to further expand the toolbox for the next new NPs. Moreover, other efforts must be done in order to create an organism with a minimal genome. This would mean for an organism to display reduced genome complexity and be engineered to have all the necessary production chassis for NP production. Filamentous fungi harness the capacity to produce a multitude of different NPs, they just need to be discovered. The future is very bright and since there is a need for novel bioactive compounds, because of the ever-present antibiotic resistance problem that is being seen, these particular platforms are of utmost importance.

### Conflict of Interest Statement

The authors declare that the research was conducted in the absence of any commercial or financial relationships that could be construed as a potential conflict of interest.

## References

[B1] Anyaogu D. C. Mortensen U. H. (2015). Heterologous production of fungal secondary metabolites in *Aspergilli*. Front. Microbiol. 6:77. 10.3389/fmicb.2015.00077 25713568 PMC4322707

[B2] Bartoszewska M. Opaliński L. Veenhuis M. van der Klei I. J. (2011). The significance of peroxisomes in secondary metabolite biosynthesis in filamentous fungi. Biotechnol. Lett. 33, 1921–1931. 10.1007/s10529-011-0664-y 21660569 PMC3173629

[B3] Bergmann S. Schümann J. Scherlach K. Lange C. Brakhage A. A. Hertweck C. (2007). Genomics-driven discovery of PKS-NRPS hybrid metabolites from *Aspergillus nidulans*. Nat. Chem. Biol. 3, 213–217. 10.1038/nchembio869 17369821

[B4] Brakhage A. A. (2013). Regulation of fungal secondary metabolism. Nat. Rev. Microbiol. 11, 21–32. 10.1038/nrmicro2916 23178386

[B5] Calcott M. J. Ackerley D. F. (2014). Genetic manipulation of non-ribosomal peptide synthetases to generate novel bioactive peptide products. Biotechnol. Lett. 36, 2407–2416. 10.1007/s10529-014-1642-y 25214216

[B6] Cameron D. E. Bashor C. J. Collins J. J. (2014). A brief history of synthetic biology. Nat. Rev. Microbiol. 12, 381–390. 10.1038/nrmicro3239 24686414

[B7] Cereghino J. L. Cregg J. M. (2000). Heterologous protein expression in the methylotrophic yeast *Pichia pastoris*. FEMS Microbiol. Rev. 24, 45–66. 10.1111/j.1574-6976.2000.tb00532.x 10640598

[B8] Chiang Y. M. Oakley B. R. Keller N. P. Wang C. C. (2010). Unraveling polyketide synthesis in members of the genus *Aspergillus*. Appl. Microbiol. Biotechnol. 86, 1719–1736. 10.1007/s00253-010-2525-3 20361326 PMC3110678

[B9] Chiang Y.-M. Oakley C. E. Ahuja M. Entwistle R. Schultz A. Chang S. L. (2013). An efficient system for heterologous expression of secondary metabolite genes in *Aspergillus nidulans*. J. Am. Chem. Soc. 135, 7720–7731. 10.1021/ja401945a 23621425 PMC3697937

[B10] Crawford J. M. Dancy B. C. Hill E. A. Udwary D. W. Townsend C. A. (2006). Identification of a starter unit acyl-carrier protein transacylase domain in an iterative type I polyketide synthase. Proc. Natl. Acad. Sci. U.S.A. 103, 16728–16733. 10.1073/pnas.0604112103 17071746 PMC1636523

[B11] Crawford J. M. Korman T. P. Labonte J. W. Vagstad A. L. Hill E. A. Kamari-Bidkorpeh O. (2009). Structural basis for biosynthetic programming of fungal aromatic polyketide cyclization. Nature 461, 1139–1143. 10.1038/nature08475 19847268 PMC2872118

[B12] Crawford J. M. Thomas P. M. Scheerer J. R. Vagstad A. L. Kelleher N. L. Townsend C. A. (2008). Deconstruction of iterative multidomain polyketide synthase function. Science 320, 243–246. 10.1126/science.1154711 18403714 PMC2480491

[B13] Crawford J. M. Townsend C. A. (2010). New insights into the formation of fungal aromatic polyketides. Nat. Rev. Microbiol. 8, 879–889. 10.1038/nrmicro2465 21079635 PMC3181163

[B14] Doudna J. A. Charpentier E. (2014). Genome editing. The new frontier of genome engineering with CRISPR-Cas9. Science 346, 1258096. 10.1126/science.1258096 25430774

[B15] Fisch K. M. Bakeer W. Yakasai A. A. Song Z. Pedrick J. Wasil Z. (2011). Rational domain swaps decipher programming in fungal highly reducing polyketide synthases and resurrect an extinct metabolite. J. Am. Chem. Soc. 133, 16635–16641. 10.1021/ja206914q 21899331

[B16] Gao L. Cai M. Shen W. Xiao S. Zhou X. Zhang Y. (2013). Engineered fungal polyketide biosynthesis in *Pichia pastoris*: a potential excellent host for polyketide production. Microb. Cell Fact. 12, 77. 10.1186/1475-2859-12-77 24011431 PMC3847973

[B17] Gressler M. Hortschansky P. Geib E. Brock M. (2015). A new high-performance heterologous fungal expression system based on regulatory elements from the *Aspergillus terreus* terrein gene cluster. Front. Microbiol. 6:184. 10.3389/fmicb.2015.00184 25852654 PMC4360782

[B18] Gründlinger M. Yasmin S. Lechner B. E. Geley S. Schrettl M. Hynes M. (2013). Fungal siderophore biosynthesis is partially localized in peroxisomes. Mol. Microbiol. 88, 862–875. 10.1111/mmi.12225 23617799 PMC3709128

[B19] Hashimoto M. Wakana D. Ueda M. Kobayashi D. Goda Y. Fujii I. (2015). Product identification of non-reducing polyketide synthases with C-terminus methyltransferase domain from *Talaromyces stipitatus* using *Aspergillus oryzae* heterologous expression. Bioorg. Med. Chem. Lett. 25, 1381–1384. 10.1016/j.bmcl.2015.02.057 25770780

[B20] Helmschrott C. Sasse A. Samantaray S. Krappmann S. Wagener J. (2013). Upgrading fungal gene expression on demand: improved systems for doxycycline-dependent silencing in *Aspergillus fumigatus*. Appl. Environ. Microbiol. 79, 1751–1754. 10.1128/AEM.03626-12 23275515 PMC3591957

[B21] Heneghan M. N. Yakasai A. A. Halo L. M. Song Z. Bailey A. M. Simpson T. J. (2010). First heterologous reconstruction of a complete functional fungal biosynthetic multigene cluster. Chembiochem 11, 1508–1512. 10.1002/cbic.201000259 20575135

[B22] Herr A. Fischer R. (2014). Improvement of *Aspergillus nidulans* penicillin production by targeting AcvA to peroxisomes. Metab. Eng. 25, 131–139. 10.1016/j.ymben.2014.07.002 25043338

[B23] Hertweck C. (2009). The biosynthetic logic of polyketide diversity. Angew. Chem. Int. Ed. Engl. 48, 4688–4716. 10.1002/anie.200806121 19514004

[B24] Hertweck C. (2015). Decoding and reprogramming complex polyketide assembly lines: prospects for synthetic biology. Trends Biochem. Sci. 40, 189–199. 10.1016/j.tibs.2015.02.001 25757401

[B25] Inglis D. O. Binkley J. Skrzypek M. S. Arnaud M. B. Cerqueira G. C. Shah P. (2013). Comprehensive annotation of secondary metabolite biosynthetic genes and gene clusters of *Aspergillus nidulans*, *A. fumigatus*, *A. niger* and *A. oryzae*. BMC Microbiol. 13:91. 10.1186/1471-2180-13-91 23617571 PMC3689640

[B26] Kakule T. B. Jadulco R. C. Koch M. Janso J. E. Barrows L. R. Schmidt E. W. (2014a). Native promoter strategy for high-yielding synthesis and engineering of fungal secondary metabolites. ACS Synth. Biol. 4, 625–633. 10.1021/sb500296p 25226362 PMC4487227

[B27] Kakule T. B. Lin Z. Schmidt E. W. (2014b). Combinatorialization of fungal polyketide synthase-peptide synthetase hybrid proteins. J. Am. Chem. Soc. 136, 17882–17890. 10.1021/ja511087p 25436464

[B28] Kealey J. T. Liu L. Santi D. V. Betlach M. C. Barr P. J. (1998). Production of a polyketide natural product in nonpolyketide-producing prokaryotic and eukaryotic hosts. Proc. Natl. Acad. Sci. U.S.A. 95, 505–509. 9435221 10.1073/pnas.95.2.505 PMC18449

[B29] Kistler H. C. Broz K. (2015). Cellular compartmentalization of secondary metabolism. Front. Microbiol. 6:68. 10.3389/fmicb.2015.00068 25709603 PMC4321598

[B30] König C. C. Scherlach K. Schroeckh V. Horn F. Nietzsche S. Brakhage A. A. (2013). Bacterium induces cryptic meroterpenoid pathway in the pathogenic fungus *Aspergillus fumigatus*. Chembiochem 14, 938–942. 10.1002/cbic.201300070 23649940

[B31] Liu T. Chiang Y. M. Somoza A. D. Oakley B. R. Wang C. C. (2011). Engineering of an “unnatural” natural product by swapping polyketide synthase domains in *Aspergillus nidulans*. J. Am. Chem. Soc. 133, 13314–13316. 10.1021/ja205780g 21815681 PMC3350771

[B32] Liu T. Sanchez J. F. Chiang Y. M. Oakley B. R. Wang C. C. (2014). Rational domain swaps reveal insights about chain length control by ketosynthase domains in fungal nonreducing polyketide synthases. Org. Lett. 16, 1676–1679. 10.1021/ol5003384 24593241 PMC3993715

[B33] Lubertozzi D. Keasling J. D. (2009). Developing *Aspergillus* as a host for heterologous expression. Biotechnol. Adv. 27, 53–75. 10.1016/j.biotechadv.2008.09.001 18840517

[B34] Ma S. M. Li J. W. Choi J. W. Zhou H. Lee K. K. Moorthie V. A. (2009). Complete reconstitution of a highly reducing iterative polyketide synthase. Science 326, 589–592. 10.1126/science 19900898 PMC2875069

[B35] Macheleidt J. Scherlach K. Neuwirth T. Schmidt-Heck W. Straßburger M. Spraker J. (2015). Transcriptome analysis of cyclic AMP-dependent protein kinase A-regulated genes reveals the production of the novel natural compound fumipyrrole by *Aspergillus fumigatus*. Mol. Microbiol. 96, 148–162. 10.1111/mmi.12926 25582336 PMC4425693

[B36] Netzker T. Fischer J. Weber J. Mattern D. J. König C. C. Valiante V. (2015). Microbial communication leading to the activation of silent fungal secondary metabolite gene clusters. Front. Microbiol. 6:299. 10.3389/fmicb.2015.00299 25941517 PMC4403501

[B37] Newman A. G. Vagstad A. L. Storm P. A. Townsend C. A. (2014). Systematic domain swaps of iterative, nonreducing polyketide synthases provide a mechanistic understanding and rationale for catalytic reprogramming. J. Am. Chem. Soc. 36, 7348–7362. 10.1021/ja5007299 24815013 PMC4046768

[B38] Nielsen M. T. Nielsen J. B. Anyaogu D. C. Holm D. K. Nielsen K. F. Larsen T. O. (2013). Heterologous reconstitution of the intact geodin gene cluster in *Aspergillus nidulans* through a simple and versatile PCR based approach. PLoS ONE 8:e72871. 10.1371/journal.pone.0072871 24009710 PMC3751827

[B39] Nützmann H. W. Fischer J. Scherlach K. Hertweck C. Brakhage A. A. (2013). Distinct amino acids of histone H3 control secondary metabolism in *Aspergillus nidulans*. Appl. Environ. Microbiol. 79, 6102–61029. 10.1128/AEM.01578-13 23892751 PMC3811361

[B40] Nützmann H. W. Reyes-Dominguez Y. Scherlach K. Schroeckh V. Horn F. Gacek A. (2011). Bacteria-induced natural product formation in the fungus *Aspergillus nidulans* requires Saga/Ada-mediated histone acetylation. Proc. Natl. Acad. Sci. U.S.A. 108, 14282–14287. 10.1073/pnas.1103523108 21825172 PMC3161617

[B41] Opalinski L. Kiel J. A. Williams C. Veenhuis M. van der Klei I. J. (2011). Membrane curvature during peroxisome fission requires Pex11. EMBO J. 30, 5–16. 10.1038/emboj.2010.299 21113128 PMC3020119

[B42] Pel H. J. de Winde J. H. Archer D. B. Dyer P. S. Hofmann G. Schaap P. J. (2007). Genome sequencing and analysis of the versatile cell factory *Aspergillus niger* CBS 513.88. Nat. Biotechnol. 25, 221–231. 10.1038/nbt1282 17259976

[B43] Podust L. M. Sherman D. H. (2012). Diversity of P450 enzymes in the biosynthesis of natural products. Nat. Prod. Rep. 29, 1251–1266. 10.1039/c2np20020a 22820933 PMC3454455

[B44] Punt P. J. Dingemanse M. A. Kuyvenhoven A. Soede R. D. Pouwels P. H. van den Hondel C. A. (1990). Functional elements in the promoter region of the *Aspergillus nidulans* gpdA gene encoding glyceraldehyde-3-phosphate dehydrogenase. Gene 93, 101–109. 2121607 10.1016/0378-1119(90)90142-e

[B45] Reverberi M. Punelli M. Smith C. A. Zjalic S. Scarpari M. Scala V. (2012). How peroxisomes affect aflatoxin biosynthesis in *Aspergillus flavus*. PLoS ONE 7:e48097. 10.1371/journal.pone.0048097 23094106 PMC3477134

[B46] Richter L. Wanka F. Boecker S. Storm D. Kurt T. Vural Ö. (2014). Engineering of *Aspergillus niger* for the production of secondary metabolites. Fung. Biol. Biotech. 1, 1–13. 10.1186/s40694-014-0004-9 PMC5598268 28955446

[B47] Robinson S. L. Panaccione D. G. (2014). Heterologous expression of lysergic acid and novel ergot alkaloids in *Aspergillus fumigatus*. Appl. Environ. Microbiol. 80, 6465–6472. 10.1128/AEM.02137-14 25107976 PMC4178656

[B48] Rugbjerg P. Naesby M. Mortensen U. H. Frandsen R. J. (2013). Reconstruction of the biosynthetic pathway for the core fungal polyketide scaffold rubrofusarin in *Saccharomyces cerevisiae*. Microb. Cell Fact. 12, 31. 10.1186/1475-2859-12-31 23557488 PMC3654996

[B49] Sakai K. Kinoshita H. Nihira T. (2012). Heterologous expression system in *Aspergillus oryzae* for fungal biosynthetic gene clusters of secondary metabolites. Appl. Microbiol. Biotechnol. 93, 2011–2022. 10.1007/s00253-011-3657-9 22083274

[B50] Sakai K. Kinoshita H. Shimizu T. Nihira T. (2008). Construction of a citrinin gene cluster expression system in heterologous *Aspergillus oryzae*. J. Biosci. Bioeng. 106, 466–472. 10.1263/jbb.106.466 19111642

[B51] Sarikaya-Bayram Ö. Palmer J. M. Keller N. P. Braus G. H. Bayram Ö. (2015). One Juliet and four Romeos: VeA and its methyltransferases. Front. Microbiol. 6:1. 10.3389/fmicb.2015.00001 25653648 PMC4299510

[B52] Schroeckh V. Scherlach K. Nützmann H. W. Shelest E. Schmidt-Heck W. Schuemann J. (2009). Intimate bacterial–fungal interaction triggers biosynthesis of archetypal polyketides in *Aspergillus nidulans*. Proc. Natl. Acad. Sci. U.S.A. 106, 14558–14563. 10.1073/pnas.0901870106 19666480 PMC2732885

[B53] Schümann J. Hertweck C. (2006). Advances in cloning, functional analysis and heterologous expression of fungal polyketide synthase genes. J. Biotechnol. 124, 690–703. 10.1016/j.jbiotec.2006.03.046 16716432

[B54] Siddiqui M. S. Thodey K. Trenchard I. Smolke C. D. (2012). Advancing secondary metabolite biosynthesis in yeast with synthetic biology tools. FEMS Yeast Res. 12, 144–170. 10.1111/j.1567-1364.2011.00774.x 22136110

[B55] Smith D. Burnham M. Edwards J. Earl A. Turner G. (1990). Cloning and heterologous expression of the penicillin biosynthetic gene cluster from *Penicillium chrysogenum*. Nat. Biotechnol. 8, 39–41. 10.1038/nbt0190-39 1368505

[B56] Spellig T. Bottin A. Kahmann R. (1996). Green fluorescent protein (GFP) as a new vital marker in the phytopathogenic fungus *Ustilago maydis*. Mol. Gen. Genet. 252, 503–509. 8914511 10.1007/BF02172396

[B57] Spröte P. Brakhage A. A. Hynes M. J. (2009). Contribution of peroxisomes to penicillin biosynthesis in *Aspergillus nidulans*. Eukaryot. Cell 8, 421–423. 10.1128/EC.00374-08 19151327 PMC2653248

[B58] Szybalski W. Skalka A. (1978). Nobel prizes and restriction enzymes. Gene 4, 181–182. 744485 10.1016/0378-1119(78)90016-1

[B59] Unkles S. E. Valiante V. Mattern D. J. Brakhage A. A. (2014). Synthetic biology tools for bioprospecting of natural products in eukaryotes. Chem. Biol. 21, 502–508. 10.1016/j.chembiol.2014.02.010 24631120

[B60] Vagstad A. L. Newman A. G. Storm P. A. Belecki K. Crawford J. M. Townsend C. A. (2013). Combinatorial domain swaps provide insights into the rules of fungal polyketide synthase programming and the rational synthesis of non-native aromatic products. Angew. Chem. Int. Ed. Engl. 52, 1718–1721. 10.1002/anie.201208550 23283670 PMC3810244

[B61] Valiante V. Macheleidt J. Föge M. Brakhage A. A. (2015). The *Aspergillus fumigatus* cell wall integrity signaling pathway: drug target, compensatory pathways, and virulence. Front. Microbiol. 6:325. 10.3389/fmicb.2015.00325 25932027 PMC4399325

[B62] Williams G. I. (2013). Engineering polyketide synthases and nonribosomal peptide synthetases. Curr. Opin. Struct. Biol. 23, 603–612. 10.1016/j.sbi.2013.06.012 23838175 PMC4271453

[B63] Wunsch C. Mundt K. Li S. M. (2015). Targeted production of secondary metabolites by coexpression of non-ribosomal peptide synthetase and prenyltransferase genes in *Aspergillus*. Appl. Microbiol. Biotechnol. 99, 4213–4223. 10.1007/s00253-015-6490-8 25744649

[B64] Xu W. Cai X. Jung M. E. Tang Y. (2010). Analysis of intact and dissected fungal polyketide synthase-nonribosomal peptide synthetase in vitro and in *Saccharomyces cerevisiae*. J. Am. Chem. Soc. 132, 13604–13607. 10.1021/ja107084d 20828130 PMC2950873

[B65] Yalpani N. Altier D. J. Barbour E. Cigan A. L. Scelonge C. J. (2001). Production of 6-methylsalicylic acid by expression of a fungal polyketide synthase activates disease resistance in tobacco. Plant Cell 13, 1401–1409. 10.1105/tpc.13.6.1401 11402168 PMC135576

[B66] Yin W. Keller N. P. (2011). Transcriptional regulatory elements in fungal secondary metabolism. J. Microbiol. 49, 329–339. 10.1007/s12275-011-1009-1 21717315 PMC3714018

[B67] Yin W.-B. Chooi Y. H. Smith A. R. Cacho R. A. Hu Y. White T. C. (2013). Discovery of cryptic polyketide metabolites from dermatophytes using heterologous expression in *Aspergillus nidulans*. ACS Synth. Biol. 2, 629–634. 10.1021/sb400048b 23758576 PMC3795930

[B68] Zabala A. O. Chooi Y. H. Choi M. S. Lin H. C. Tang Y. (2014). Fungal polyketide synthase product chain-length control by partnering thiohydrolase. ACS Chem. Biol. 9, 1576–1586. 10.1021/cb500284t 24845309 PMC4215887

[B69] Zadra I. Abt B. Parson W. Haas H. (2000). xylP promoter-based expression system and its use for antisense downregulation of the *Penicillium chrysogenum* nitrogen regulator NRE. Appl. Environ. Microbiol. 66, 4810–4816. 10.1128/AEM.66.11.4810-4816.2000 11055928 PMC92384

[B70] Zhu X. Yu F. Bojja R. S. Zaleta-Rivera K. Du L. (2006). Functional replacement of the ketosynthase domain of *FUM1* for the biosynthesis of fumonisins, a group of fungal reduced polyketides. J. Ind. Microbiol. Biotechnol. 33, 859–868. 10.1007/s10295-006-0137-9 16683125

[B71] Zhu X. Yu F. Li X. C. Du L. (2007). Production of dihydroisocoumarins in *Fusarium verticillioides* by swapping ketosynthase domain of the fungal iterative polyketide synthase Fum1p with that of lovastatin diketide synthase. J. Am. Chem. Soc. 129, 36–37. 10.1021/ja0672122 17199276

